# Molecular and cellular neuroinflammatory status of mouse brain after systemic lipopolysaccharide challenge: importance of CCR2/CCL2 signaling

**DOI:** 10.1186/1742-2094-11-132

**Published:** 2014-07-28

**Authors:** Julie Cazareth, Alice Guyon, Catherine Heurteaux, Joëlle Chabry, Agnès Petit-Paitel

**Affiliations:** 1 Université de Nice Sophia Antipolis, Nice 06103, France; 2 Centre National de la Recherche Scientifique (CNRS), Institut de Pharmacologie Moléculaire et Cellulaire, Valbonne 06560 UMR 7275, France; 3 IPMC-CNRS UMR 7275, 660 Route des Lucioles, Valbonne 06560, France

**Keywords:** Microglia, Monocytes, Macrophages, Neuroinflammation, Brain infiltration, Cytokines, Chemokines, CCL2, Serotonin, Depression

## Abstract

**Background:**

Genetic and environmental factors are critical elements influencing the etiology of major depression. It is now accepted that neuroinflammatory processes play a major role in neuropsychological disorders. Neuroinflammation results from the dysregulation of the synthesis and/or release of pro- and anti-inflammatory cytokines with central or peripheral origin after various insults. Systemic bacterial lipopolysaccharide (LPS) challenge is commonly used to study inflammation-induced depressive-like behaviors in rodents. In the present study, we investigated immune-to-brain communication in mice by examining the effects of peripheral LPS injection on neuroinflammation encompassing cytokine and chemokine production, microglia and central nervous system (CNS)-associated phagocyte activation, immune cell infiltration and serotonergic neuronal function.

**Methods:**

LPS was administered to C57BL/6 J mice by intraperitoneal injection; brains were collected and pro-inflammatory cytokine mRNA and proteins were measured. To examine the relative contribution of the different populations of brain immune cells to the occurrence of neuroinflammation after acute systemic inflammation, we precisely characterized them by flow cytometry, studied changes in their proportions and level of activation, and measured the amount of cytokines they released by Cytometric Bead Array™ after cell sorting and *ex vivo* culture. Because of the central role that the chemokine CCL2 seems to play in our paradigm, we studied the effect of CCL2 on the activity of serotonergic neurons of the raphe nucleus using electrophysiological recordings.

**Results:**

We report that systemic LPS administration in mice caused a marked increase in pro-inflammatory IL-1β, IL-6, TNFα and CCL2 (monocyte chemoattractant protein-1) mRNA and protein levels in the brain. Moreover, we found that LPS caused microglia and CNS-associated phagocyte activation characterized by upregulation of CCR2, TLR4/CD14, CD80 and IL-4Rα, associated with overproduction of pro-inflammatory cytokines and chemokines, especially CCL2. LPS also induced a marked and selective increase of CCR2^+^ inflammatory monocytes within the brain. Finally, we showed that CCL2 hyperpolarized serotonergic raphe neurons in mouse midbrain slices, thus probably reducing the serotonin tone in projection areas.

**Conclusion:**

Together, we provide a detailed characterization of the molecular and cellular players involved in the establishment of neuroinflammation after systemic injection of LPS. This highlights the importance of the CCL2/CCR2 signaling and suggests a possible link with depressive disorders.

## Background

Inflammation plays a central role in the pathogenesis of several brain disorders, including depression. Depression is a multifactorial disease with both genetic and environmental factors. Beyond the recognized role of monoaminergic systems including serotonin (5-HT), norephinephrine (NE) and dopamine (DA), a link between inflammatory processes and depression etiology has been firmly established at both the clinical and preclinical levels [1-8]. Depression may be due to the central action of cytokines on the hypothalamus-pituitary-adrenal (HPA) axis [9] and/or to a reduced hippocampal neurogenesis [10-12]. Several studies have reported that depressed patients commonly display alterations in their immune system, including impaired cellular immunity and elevated levels of pro-inflammatory cytokines in the plasma and cerebrospinal fluid, mainly IL-1β, IL-6 and TNFα [13-16]. Further evidence of the close link between inflammation and depression comes from clinical observations showing that patients receiving the pro-inflammatory cytokines IL-2 and interferon-α during cancer or hepatitis C treatments frequently develop major depression symptoms [17-19].

Systemic injection of the endotoxin lipopolysaccharide (LPS), a cell wall component of Gram-negative bacteria, can generate many features of the acute phase response of inflammation [20,21] and has therefore been used extensively as a model for peripherally induced inflammation. Systemic LPS causes an increase in pro-inflammatory cytokine production in circulating or tissue-resident immune cells such as monocytes and macrophages [22]. For many years, the brain has been considered as an immunologically privileged site; however, many developments in neuro-immunology have challenged this concept [23]. Indeed, in response to infection or injury, the brain exhibits many hallmarks of inflammation such as edema, activation of brain mononuclear phagocytes (microglia and macrophages), local invasion of circulating immune cells, and production of cytokines (for review, see [24]). Peripherally produced cytokines (for example, after systemic LPS challenge) may transfer an inflammatory signal to the brain in several ways: activation in areas of the blood-cerebrospinal fluid barrier such as choroid plexus, active transport of cytokines across the blood–brain barrier (BBB), or modulation of afferent nerves. Another possibility is the direct activation of certain brain cells by LPS, as some of them express the LPS receptor, namely the Toll like receptor 4 (TLR4) [25]. Moreover, migration of blood cells within the central nervous system (CNS) may also contribute to a brain inflammation state [26]. These peripheral inflammatory signals stimulate innate immune brain cells to endogenously express the same set of pro-inflammatory cytokines [20,27].

Different immune cell populations contribute to CNS neuroinflammation: CNS-resident microglia and CNS-associated mononuclear phagocytes, among which neutrophils, CNS-resident perivascular and meningeal macrophages, choroid plexus macrophages and CNS-infiltrating monocyte-derived macrophages should be distinguished [28]. However, until now, a clear vision of the precise role of each of these populations in neuroinflammation processes failed to emerge from the literature due to a lack of homogeneity in their parameters of identification and characterization.

Microglia have two main functions within the CNS: maintenance of neuronal functions and immune defense [29,30]. However, dysregulated and/or sustained activation of microglia may induce neuroinflammation and contribute to progression of almost all brain diseases, including depression [31-33]. Indeed, in experimental studies it has been shown that CCL2 activates microglia triggering the release of IL-1β and TNFα [34]. Moreover, it has been shown that a single injection of IL-1β or TNFα into selected regions of the brain results in sickness behaviors, activation of the HPA axis and inhibition of hippocampal neurogenesis, resulting in cognition impairment and depressive-like behavior in animal models [9,21]. As one of the main sources of pro-inflammmatory cytokines in the brain and since they express TLR4, microglia could be a direct target of systemic LPS challenge.

CNS-associated macrophages reside within the vascular basement membrane in close proximity to blood vessels [35,36]. They are also associated with the leptomeninges [37] and the choroid plexus [38]. In inflammatory conditions, the CNS-associated mononuclear phagocyte population also includes CNS-infiltrating monocyte-derived macrophages and neutrophils, which all could contribute to the establishment and maintenance of a neuroinflammatory state [39].

In response to insult or injury, microglia and macrophages acquire diverse and complex phenotypes, allowing them to participate in the cytotoxic response, immune regulation, and injury resolution. Nomenclature of these phenotypes can be characterized into four main states, a “classically activated” M1 state with cytotoxic properties, an “alternative activation” M2a state involved in repair and regeneration, an “immunoregulatory” M2b state, and a “deactivating phenotype” M2c state. These phenotypes have known prototypical inducers: TLR4 agonists as LPS and TNFα for the M1 state; IL-4 and IL-13 for the M2a state; immune complexes, TLR4 agonists and IL-1R ligands for the M2b state; and IL-10, transforming growth factor-β and glucocorticoids for the M2c state. However, most of the studies outlining these inducers were performed on blood-derived macrophages, while microglia are known to differ in their responsiveness to stimuli [40].

It is known that peripherally administered LPS induces an innate immune response characterized by a rapid activation of microglia and brain endothelial cells, expression of various pro-inflammatory mediators, and subsequent immune cell infiltration into the brain parenchyma [41-48], which are pathological features of many cerebral diseases such as depression. However, the precise molecular signaling molecular pathways and the contribution of the different sub-populations of brain-associated microglia/macrophages are not fully understood.

Pro-inflammatory cytokines also influence the 5-HT, NE and DA neurotransmitter systems [49]. There are several known mechanisms by which cytokines reduce serotonergic neurotransmission. For example, pro-inflammatory cytokines activate the tryptophan-kynurenine pathway whereby tryptophan is shunted from the synthesis of 5-HT to that of kynurenine [50,51]. Although this mechanism is important in serotonergic system dysfunction in depression, other possible mechanisms cannot be ruled out and remain to be elucidated.

In the present study, we investigated how systemic inflammation triggered by a single intraperitoneal (i.p.) injection of LPS affected brain inflammation, microglia and CNS-associated phagocyte features, immune cell infiltration and cytokine production profiles. The consequences of such inflammation on serotonergic neuronal dysfunction were also investigated. Altogether, our results highlight the important role of the CCL2/CCR2 signaling pathway in neuroinflammation and bring new insights regarding the mechanisms possibly involved in the onset of neuroinflammation-related depressive behaviors.

## Methods

### Animals and lipopolysaccharide administration

Eight-week-old female C57BL/6 J mice were purchased from Janvier (Saint-Quentin-Fallavier France). All mice were held in a temperature-controlled room maintained under a 12-hour light/dark cycle and had access to food and water *ad libitum*. Animals were handled in accordance with good animal practice as defined by the relevant national animal welfare bodies, equivalent to the European Convention for the Protection of Vertebrate Animals used for Experimental and other Scientific Purposes (ETS 123). Mouse experimentation protocols were approved by the Nice Sophia Antipolis University animal safety committee (CIEPAL-Azur, NCE/2011-21).

LPS from *Escherichia coli* 0111:B4 was purchased from Sigma-Aldrich (Saint-Quentin-Fallavier, France) and freshly dissolved in sterile saline prior to i.p. injection. Mice were injected i.p. with vehicle (0.9% NaCl) or LPS (2 mg/kg).

### Isolation of immune cells from adult mouse brains

Mice were deeply anesthetized 24 hours post i.p. injection with a lethal injection of pentobarbital. Immune brain cells were isolated from whole brain homogenates as follows. Mice were transcardially perfused with ice-cold PBS (pH 7.4, 1 mg/ml EDTA). Brains were collected and roughly homogenized in PBS, resuspended in PBS containing 3 mg/ml collagenase D (Roche Diagnostics, Meylan, France) and incubated for 30 minutes at 37°C. After incubation, brain homogenates were filtered in 70 μm pore size cell strainers (BD Biosciences, Le Pont de Claix, France), centrifuged (10 minutes, 2,000 rpm), washed and resuspended in 6 ml of 38% isotonic Percoll (GE Healthcare, Aulnay Sous Bois, France), before centrifugation (20 minutes, 2,000 rpm, 4°C). Myelin and debris were discarded. Cell pellets containing brain immune cells were collected, washed and labeled for subsequent cell sorting and/or flow cytometry analysis.

### Brain immune cell staining, flow cytometry and cell sorting

Staining of brain immune cell surface antigens was performed as follows. In brief, Fc receptors were blocked with 2.4G2 antibody. Cells were incubated with the appropriate combination of conjugated antibodies: CD11b-PercP-Cy5.5, CD45-APC-Cy7, Ly6C-PE-Cy7, Ly6G-pacific blue, CD3-FITC, CD8-APC, major histocompatibility complex class II-Alexa700, CD80-V450, CD86-eFluor605, CD14-APC, TLR4-Alexa488, CD124/IL-4Rα-Biotin and streptavidin-PE-Cy7 (BD Biosciences), CD4-Viogreen (Miltenyi Biotec, Paris, France), CCR2-PE (R&D systems, Lille, France) or isotype control antibodies for 30 minutes. Cells were washed and resuspended in PBS containing 0.5% bovine serum albumin for analysis and cell sorting with FACS Aria III (BD Biosciences).

### *Ex vivo* culture of sorted brain immune cells

Microglia and CNS-associated phagocytes were isolated from the saline- and LPS-treated brain of 8-week-old mice as described above, and seeded at a density of 3 × 10^4^ cells/well in 96-well tissue culture plates (Falcon, Greiner, Courtaboeuf, France) in Dulbecco's modified Eagle's medium culture media (Invitrogen, Cergy Pontoise, France) containing 10% fetal bovine serum. Cells were cultured at 37°C with 5% CO_2_ and saturated humidity for 24 hours with or without stimulation with 0.5 μg/ml LPS. Media were then collected and centrifuged at 1,200 rpm for 10 minutes at 4°C. The supernatants were used for Cytometric Bead Array™ (CBA; BD Biosciences) measurement of cytokines and chemokines.

### Cytokine measurement by cytometric bead array

Selected brain regions from saline- or LPS-injected brains were homogenized in NP-40 containing buffer (10 mM Tris–HCl pH 8.0, 150 mM NaCl, 1% NP-40, 10% glycerol, 5 mM EDTA and protease inhibitor cocktail (Roche Diagnostics)) according to Amsen and colleagues [52]. Supernatants from brain homogenates or *ex vivo* cultured cells were harvested and the concentration of secreted cytokines (TNFα, CCL2, IL-1β and IL-6) was detected using a CBA according to the manufacturer’s instructions (BD Biosciences). For comparison, data were normalized relative to the protein concentration of related brain homogenate, or alternatively to the cell number.

### Immunohistochemistry

To stain microglia/macrophages, mice were deeply anesthetized with pentobarbital and transcardially perfused with PBS and then 3.2% paraformaldehyde. Brains were postfixed in 3.2% paraformaldehyde for 24 hours and incubated in 20% sucrose for an additional 24 hours. Fixed brains were frozen using isopentane (−30°C) and sectioned (20 μm) using a cryostat. Brain regions were identified by reference markers in accordance with the stereotaxic mouse brain atlas. Sections were mounted on slides and blocked with 5% goat serum. Sections were washed and incubated with a rabbit anti-mouse ionized calcium binding adaptor molecule 1 (Iba-1) antibody (Abcam, Paris, France) overnight at 4°C. After washes, the sections were incubated with secondary biotinylated goat anti-mouse IgG and DAB staining was performed according to manufacturer’s instructions (DAB peroxidase substrate kit, Vector Laboratories, Clinisciences, Nanterre, France).

### RNA isolation and quantitative polymerase chain reaction

Total RNA from saline- or LPS-injected brain areas, or from brain immune cells purified and sorted according to CD11b and CD45 surface labeling were isolated using the Trizol® RNA extraction kit (Invitrogen) according to the manufacturer recommendations followed by a RQ1 DNAse (Promega, Charbonnières, France) treatment. First-strand cDNA were synthesized from 2 μg of total RNA with 200 U of SuperScript III reverse transcriptase (SuperScriptIII, Invitrogen) in the appropriate buffer in the presence of 25 μg/ml random primers, 0.5 mM desoxyribonucleotide triphosphate mix, 5 mM dithiothreitol, 40 U RNAsin (Promega). The reaction was incubated for 5 minutes at 25°C, then 50 minutes at 50°C, and then inactivated for 15 minutes at 70°C. Quantitative PCR was performed using the SYBRgreen method (Roche, Boulogne-Billancourt, France) with the LightCycler 480 sequence detector system (Roche Diagnostics). Primers were purchased from QIAGEN (QuantiTect primer assay, QIAGEN, Courtaboeuf, France).

### Brain slices for electrophysiological recordings

Female mice (12 to 27 days old) were anaesthetized with 1% halothane. Following decapitation, brains were rapidly removed and placed in cold phosphate/bicarbonate buffered solution (PBBS, 4°C) composed of (mM): 125 NaCl, 2.5 KCl, 0.4 CaCl_2_, 1 MgCl_2_, 25 glucose, 1.25 NaH_2_PO_4_, 26 NaHCO_3_, pH 7.4 when bubbled with 95% O_2_/5% CO_2_. Transversal 250 μm thick slices cut with a vibrating microtome (Microm, Francheville, France) were then transferred to an incubating chamber maintained at 34°C in oxygenated PBBS. After 1 hour, slices were transferred to another incubating chamber at room temperature filled with PBBS containing additional CaCl_2_ (final concentration, 2 mM).

### Patch clamp technique

Mice brain slices containing the raphe nucleus were placed under a Nomarski microscope (Zeiss, Marly Le Roi, France) equipped with an infrared video camera (Axiocam, Zeiss) in a recording chamber superfused at a flow rate of 1 ml/minute with oxygenated PBBS (2 mM CaCl_2_). Recordings from median and dorsal raphe nucleus neurons were made at room temperature (25 ± 2°C) using a Axopatch 200B (Axon Instruments, Union City, USA). Patch clamp pipettes made from borosilicate glass capillary (Hilgenberg, Malsfeld, Germany) had a resistance of 2 to 8 MΩ when filled with the internal solution containing (mM): 130 Kgluconate, 1 MgCl_2_, 0.3 CaCl_2_, 1 EGTA, 4 Mg2ATP, 0.4 Na3GTP, 10 HEPES (pH adjusted to 7.3 with KOH). Neurons were first patch-clamped in cell-attached mode to record spontaneous action potential firing and then in the whole-cell configuration in current clamp. Values of access resistance ranged from 12 to 20 MΩ and were not compensated. Measurements were made 2 to 3 minutes after obtaining the whole cell to ensure dialysis. Cell capacitance and resistance were measured in voltage clamp using the pClamp Clampex software (Axon Instruments, Union City, USA) by applying 5 mV voltage steps. Solutions were applied in the bath. Current-clamp recordings were made in the IClamp mode of the Axopatch 200B.

Voltage clamp data were digitized at 0.5 kHz using a Digidata interface coupled to a microcomputer running p-Clamp 9 (Axon Instruments).

All electrophysiological chemicals were from Sigma-Aldrich.

### Statistical analysis

Statistical analysis was performed using Statistica software™ and SigmaStat 2.03 (Jandel Sci, Castets, France). Values are reported as mean ± SEM. Significant differences between two groups of data were determined using a *t* test for continuous data, meeting parametric assumptions of equal variance and normality. Otherwise, a Mann–Whitney test was performed for nonparametric data.

## Results

### Peripheral lipopolysaccharide administration induced M1-type pro-inflammatory cytokine/chemokine mRNA and protein profiles in mouse brain

In a first set of studies we determined the expression of pro-inflammatory cytokine and chemokine genes in three distinct brain regions (that is, hypothalamus, hippocampus and the choroid plexus). We chose these regions because they all are implicated, although in different ways, in neuroinflammatory processes. Indeed, peripheral LPS challenge induces activation of the HPA axis and causes central cytokine production in the CNS that is responsible for sickness and depressive behaviors. The central pro-inflammatory cytokines are responsible for the reduction of hippocampal neurogenesis that has been implicated in depression. Regarding the brain’s choroid plexus, this complex structure is the source of cerebrospinal fluid and it might also be a portal of entry for immune cells to invade the CNS and initiate neuroinflammation and disorders arising therefrom.

Adult female mice were sacrificed 24 hours after peripheral LPS injection and brains were collected. Table 1 shows the relative mRNA expression of the pro-inflammatory cytokines IL-1β, IL-6, TNFα and CCL2 chemokine in the hypothalamus, hippocampus and the choroid plexus. As expected, LPS increased the mRNA of all pro-inflammatory genes in the three examined regions, to various extents. TNFα mRNA levels were dramatically increased in all considered brain areas, especially in the choroid plexus. IL-1β, IL-6 and CCL2 mRNA were highly elevated in the hippocampus, while the increases in the hypothalamus and the choroid plexus were modest (Table 1).

**Table 1:** Peripheral lipopolysaccharide administration induced M1-type pro-inflammatory cytokine/chemokine mRNA profiles in mouse brain

**Gene expression (fold increase 24 hours post-intraperitoneal LPS)**				
	**IL-1β**	**IL-6**	**TNFα**	**CCL2**
Hypothalamus	6.40 ± 0.79**	2.18 ± 0.42*	22.51 ± 1.09***	1.33 ± 0.72*
Hippocampus	23.02 ± 0.30***	17.88 ± 1.22*	77.71 ± 0.74***	24.01 ± 1.74*
Choroid plexus	6.93 ± 0.78**	2.29 ± 0.98*	1477.8 ± 1.25***	2.29 ± 0.98 (ns)

Adult 8-week-old C57BL/6 J female mice were injected intraperitoneally with saline (0.9% NaCl) or 2 mg/kg lipopolysaccharide (LPS). Brains were dissected 24 hours later and pro-inflammatory genes for IL-1β, IL-6, TNFα and CCL2 were determined by quantitative PCR from the hypothalamus, hippocampus and choroid plexus. *P < 0.05, **P < 0.01, ***P < 0.005 versus saline controls; ns, not significant, n = 4.

**Figure 1:**
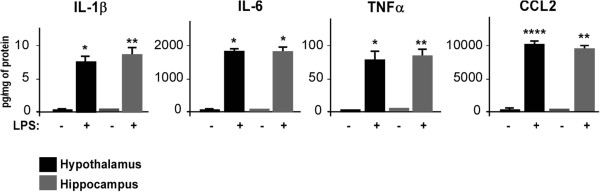
**Peripheral lipopolysaccharide administration induces M1-type pro-inflammatory cytokine/chemokine protein profiles in mouse brain.** Mice were injected intraperitoneally with saline or 2 mg/kg lipopolysaccharide (LPS). Brains were removed 24 hours later, and the hypothalamus and hippocampus were dissected and homogenized. The pro-inflammatory cytokines IL-1β, IL-6, TNFα and chemokine CCL2 were quantified by cytometric bead array. Bars represent the mean ± SEM. *P < 0.05, **P < 0.01, ***P < 0.005, ****P < 0.001 versus saline control; n = 5.

The protein amount was also assayed by CBA 24 hours after injection in both the hypothalamus and hippocampus from saline- and LPS-treated mice (Figure 1). LPS induced an increase in all measured pro-inflammatory cytokines and chemokines to a similar extent in the hypothalamus and hippocampus. Indeed, in both brain areas the CCL2 amount was dramatically increased by LPS (95.69 ± 7.51 pg/mg versus 9,925.44 ± 1,411.33 pg/mg in saline- and LPS-treated hypothalamus, respectively, *P* < 0.01; 33.34 ± 7.51 pg/mg versus 8,629.46 ± 545.68 pg/mg in saline- versus LPS-treated hippocampus, respectively, *P* < 0.001; Figure 1). In saline-treated brains, no or very low levels of cytokines and chemokines were detectable and LPS significantly increased the amounts of IL-1β, IL-6 and TNFα (for IL-1β: not detectable versus 6.90 ± 1.00 pg/mg in saline- and LPS-treated mice hypothalamus, respectively, *P* < 0.05; not detectable versus 8.48 ± 2.00 pg/mg in saline- and LPS-treated mice hippocampus, respectively, *P* <0.01, for IL-6: 14.37 ± 6.20 pg/mg versus 1,716.584 ± 326.52 pg/mg in saline- and LPS-treated mice hypothalamus, respectively, *P* < 0.05; 3.21 ± 1.21 pg/mg versus 1,784.19 ± 211.34 pg/mg in saline- versus LPS-treated mice hippocampus, respectively, *P* < 0.05; and for TNFα: not detectable versus 74.88 ± 10.85 pg/mg in saline- and LPS-treated mice hypothalamus, respectively, *P* < 0.05; not detectable versus 81.76 ± 29.14 pg/mg in saline- versus LPS-treated mice hippocampus, respectively, *P* < 0.01; Figure 1).

### Peripheral lipopolysaccharide induced an increase in central nervous system-associated phagocytes in mouse brain

**Figure 2:**
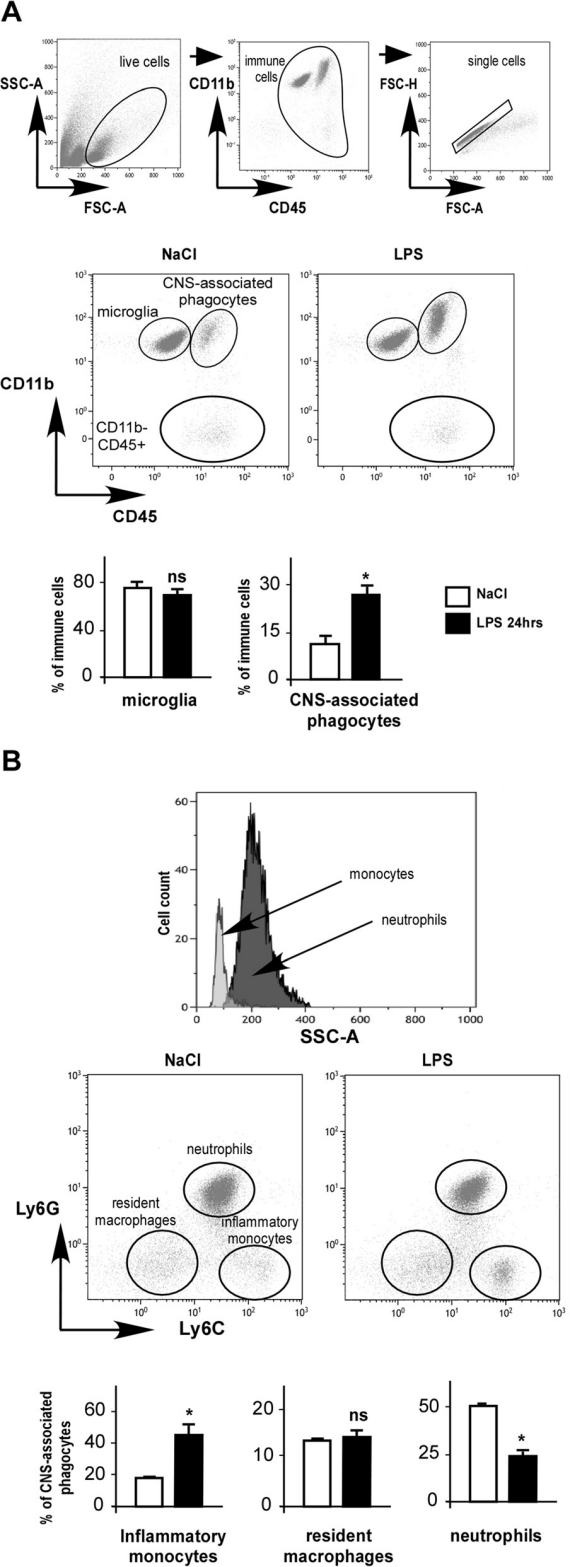
**Peripheral lipopolysaccharide induces increases in inflammatory monocytes in mouse brain.** Adult (8 weeks old) C57BL/6 female mice were injected intraperitoneally with saline or 2 mg/kg lipopolysaccharide (LPS), and CD11b, CD45, Ly6C, Ly6G expressions were determined by flow cytometry from brain cell suspensions isolated 24 hours later. **(A)** Upper and middle panels show representative bivariate dot plots of Percoll isolated brain cells illustrating a gating strategy to exclude dead cells, debris and aggregated cells, and to determine profiles based on CD11b^+^/CD45^low+^ for microglia, CD11b^+^/CD45^high+^ for central nervous system (CNS)-associated phagocytes and CD11b^−^/CD45^high+^ cells. Lower panels show histograms of average percentage of microglia (left), CNS-associated phagocytes (right) in live single immune cells from brains of saline- (white bars) and LPS-injected mice (black bars). **(B)** Upper panel shows side scatter (SSC) plot. This shows that, among CNS-associated phagocytes, macrophages/monocytes and neutrophils can be distinguished according to their granulosity. Middle panels show representative bivariate dot plots of phagocytes stained for Ly6G and Ly6C illustrating a gating strategy to identify Ly6C^intermediate+^/Ly6G^high+^ neutrophils, Ly6C^−^/Ly6G^−^ resident macrophages and Ly6C^high+^/Ly6G^−^ inflammatory monocytes. Lower panels show histograms of average percentage of inflammatory monocytes (left), resident macrophages (middle) and neutrophils (right) in CNS-associated phagocytes population from brains of saline (white bars) and LPS mice (black bars). Bars represent the mean ± SEM. *P < 0.05 versus saline control; ns, non significant; n = 4. FSC, front scatter.

Distinguishing CNS-inflammatory monocytes, resident macrophages and microglia based on differential antigen expression is almost impossible using conventional immunohistochemical techniques, but it can be accomplished using specific surface marker labeling coupled to a detailed flow cytometry analysis. Indeed, microglia and other monocytes can essentially be identified based on their CD11b and CD45 expression levels. Microglia express high levels of CD11b and low levels of CD45 (CD11b^+^/CD45^low+^) while the heterogeneous population of CNS-associated phagocytes including brain macrophages express high levels of both CD11b and CD45 (CD11b^+^/CD45^high+^). In the front scatter and side scatter (SSC) plots, different cell populations and cell debris were identified in our brain cell suspensions. Dead cells, debris and cell doublets were excluded, and a live single immune cell gate was created and used for further analysis (Figure 2A, upper panels). Three different populations were identified in this gate: CD11b^+^/CD45^high+^ (CNS-associated phagocytes), CD11b^+^/CD45^low+^ (microglia), and CD11b^−^/CD45 ^high+^ cells (Figure 2A, middle panels).

In brains from LPS-treated mice, the percentage of CD11b^+^/CD45^high+^ cells was significantly higher compared to brains from saline-treated mice (25.12 ± 3.08% in LPS-treated brain versus 12.98 ± 3.71 in saline-treated brain, *P* < 0.05; Figure 2A, histograms). Notably, the ratio of microglia/CNS-associated phagocytes is dramatically different in these two conditions, being much lower after LPS challenge (2.52 ± 0.94 in LPS-treated brain versus 6.52 ± 2.32 in saline-treated brain, *P* < 0.05; data not shown). This may indicate a proliferation of resident macrophages and/or a higher number of infiltrating cells from the periphery. Our data does not show significant proliferation of microglia upon LPS treatment (Figure 2A). However, microglia proliferation might have been masked by the dramatic increase of CNS-associated phagocytes in the brain or might occur at a different time after LPS challenge.

To further characterize which cell types migrate from the periphery to the brain in cases of inflammation, we performed detailed analysis of CD11b^+^/CD45^high+^ cells by flow cytometry. Ly6C and Ly6G surface marker labeling allowed us to discriminate between three subpopulations among immune brain CD11b^+^/CD45^high+^ cells. Neutrophils were identified as CD11b^+^/CD45^high+^/Ly6C^intermediate+^/Ly6G^high+^ cells and macrophages were identified as CD11b^+^/CD45^high+^/Ly6G^neg-^ cells. Among them, resident macrophages were identified as Ly6C^neg-^ and inflammatory monocytes as Ly6C^high+^ cells (Figure 2B, middle panel). SSC plots confirmed this discrimination, since neutrophils are known to be SSC^high^ (Figure 2B, upper panel). The specificity of Ly6C and Ly6G signals was verified by labeling with isotype control antibodies (data not shown). Our results showed that peripheral LPS administration promoted a selective migration of inflammatory monocytes to the brain, 24 hours after treatment (44.70 ± 6.80% of parent cells in LPS-treated mouse brain versus 17.46 ± 0.91% in saline-treated brain, *P* < 0.05; Figure 2B, left histogram). Resident macrophage percentage was not significantly affected by LPS treatment (14.5 ± 1.66% of parent cells in LPS-treated mouse brain versus 13.7 ± 0.81% in saline-treated brain; Figure 2B, middle histogram) while neutrophil relative percentage was reduced (56.6 ± 0.33% of parent cells in saline-treated mouse brain versus 29.9 ± 3.73% in LPS-treated brain, *P* < 0.05; Figure 2B, right histogram). These results do not exclude the possibility of a proliferation of resident macrophages and/or an infiltration of neutrophils but indicate that they probably are minor events compared to the massive inflammatory monocyte increase and/or that they may occur at a different time point after LPS challenge. It is also interesting to note that a sub-population of the CNS-associated phagocytes clearly overexpressed the CD11b marker in brains of LPS-treated mice as compared to control brains (Figure 2A). Our results show that this overexpression was almost exclusively restricted to brain neutrophils, suggesting their activation by systemic LPS (data not shown).

The infiltration of lymphocytes within the brain after i.p. injection of LPS was also studied. Among the CD11b^−^/CD45^high+^ population, we compared CD3^−^/B220^+^ (B lymphocytes), B220^−^/CD3^+^/CD4^+^ expressing cells (CD4^+^ lymphocytes) and B220^−^/CD3^+^/CD8^+^ (CD8^+^ lymphocytes) expressing cells in brains from saline-treated mice and LPS-treated mice. No statistical differences were detected, indicating than in our experimental conditions peripherally administered LPS does not produce significant B or T lymphocyte infiltration within the brain, 24 hours after treatment (data not shown).

### Peripheral lipopolysaccharide induced microglia/macrophage M2b-type activation and CCR2 overexpression on both cell types

**Figure 3:**
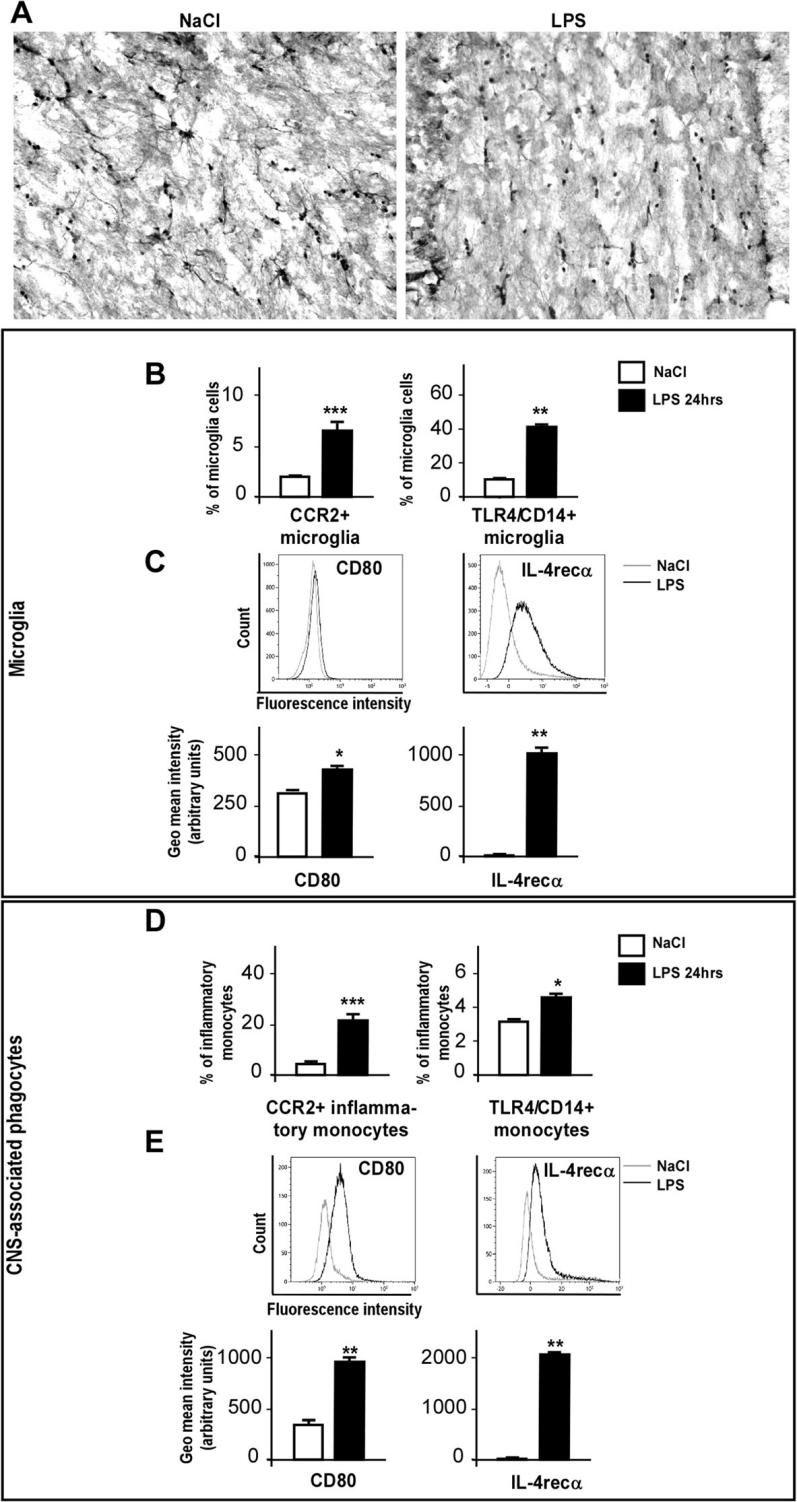
**Peripheral lipopolysaccharide induces microglia/monocyte M2b-type activation and CCR2 overexpression in both cell types.** Brain sections from saline- and lipopolysaccharide (LPS)-injected mice were stained with ionized calcium binding adaptor molecule 1 (Iba-1) antibody **(A)** (n = 3) or cells were prepared for flow cytometry analysis **(B-E)**. **(A)** Representative pictures of Iba-1 immunoreactive cells in the hippocampus of brains from saline (left) and LPS-injected (right) mice. **(B, D)** Histograms of average percentage of microglia **(B)** or inflammatory monocytes **(D)** expressing CCR2 (left) and TLR4/CD14 complex (right) in live single immune cell suspensions from brains of saline- (white bars) and LPS-injected mice (black bars). **(C,E)** Graphs and histograms of mean fluorescent intensity for CD80 (left) and CD124/IL-4Rα (right) in enriched microglia **(C)** and enriched central nervous system (CNS)-associated phagocyte **(E)** populations from brains of saline- (white bars) and LPS-treated mice (black bars). Bars represent the mean ± SEM. *P < 0.05, **P < 0.01, ***P < 0.005 versus saline control; n = 4.

Activation of peripheral macrophages through TLR4 by LPS is described to promote the M1 state. The dramatic increase of IL-1β, IL-6, TNFα and CCL2 gene expression in the hippocampus and hypothalamus of LPS-treated mice (Figure 1) suggested that LPS could cause a polarization of CNS-associated macrophages and microglia cells towards a conventional pro-inflammatory activation state of the M1 type. In order to further characterize the microglia and macrophage activation state from LPS-injected mouse brains, we performed morphological studies of both microglia and macrophages by immunohistochemistry with the Iba-1 antibody, which labels both cell types, and specific surface marker examination of each cell type by flow cytometry. Twenty-four hours after LPS i.p. injection, Iba-1 immunohistochemistry revealed that microglia/macrophages retracted their processes and enlarged their cell bodies, confirming their shift from a ‘resting’ to an ‘activated’ state (Figure 3A). We chose to examine as M1 markers the expression of the co-stimulatory molecules CD80 and CD86, the major histocompatibility complex class II, the chemokine receptor 2 (CCR2), the TLR4/CD14 complex, and IL-4Rα as M2 activation markers.

In the periphery, inflammatory monocytes express CCR2, emerge from bone marrow in response to chemokines such as CCL2, and home to sites of inflammation. In order to study whether brain CCL2 release after systemic LPS challenge could also affect CCR2 expressing cells within the brain and periphery, we examined CCR2 expression by flow cytometry on microglia and CNS-associated phagocytes in brains from control and LPS-injected mice. Figure 3B (left histogram) shows that the percentage of CCR2-expressing microglia cells increased with LPS treatment. In the population of inflammatory monocytes, CCR2 expression was higher in basal conditions as compared to microglia (5.9 ± 1.27% of inflammatory monocytes versus 2.05 ± 0.31% of microglia in brains of saline-treated mice, Figure 3B,D) and was increased to a larger extent by LPS i.p. injection (26.4 ± 2.26% of parent cells in brains of LPS-i.p. mice versus 5.8 ± 1.27 in brains of saline-treated mice, *P* < 0.005; Figure 3D, left histogram), suggesting an important role of the CCL2/CCR2 signaling pathway in attraction/migration of immune cells to the brain during inflammatory processes. Antibody specificity was checked using isotype control antibody labeling on microglia and macrophages (data not shown).

Figure 3B,D (right histograms) confirmed that microglia and CNS-associated phagocytes expressed both TLR4 receptor and CD14, and that LPS treatment increased their expression. Figure 3C,E shows that CD80 expression level was upregulated by LPS i.p. injection in both microglia and CNS-associated phagocytes. Conversely, CD86 expression was barely detectable on both cell types and was not significantly affected by LPS treatment (data not shown). In accordance with a recent publication [53], LPS treatment induced high expression levels of IL-4Rα on the surface of microglia (Figure 3C). Our results provide the additional information that CNS-associated phagocytes present in the brain after LPS i.p. also express high levels of IL-4Rα (Figure 3E, right panels). These results are consistent with the recent publication suggesting that LPS would promote a state of activation of the M1 type of microglia while making them more sensitive to the M2-promoting effects of some anti-inflammatory cytokines such as IL-4 [53]. Our results on inflammatory monocytes suggest that this cell population would acquire the same peculiar phenotype. In contrast to previous works on rat brain [54,55], no overexpression of major histocompatibility complex class II on the surface of microglia cells or CNS-associated phagocytes following treatment with LPS was observed (data not shown), suggesting that, 24 hours after LPS i.p. administration, the antigen presentation function of microglia might not be altered in our experimental model. Moreover, no expression of the dendritic marker CD11c was detected at the surface of the immune brain cells (data not shown) 24 hours after LPS i.p. injection.

### Lipopolysaccharide promoted M1-type pro-inflammatory cytokine production by microglia and central nervous system-associated phagocytes sorted from mouse brain

**Figure 4:**
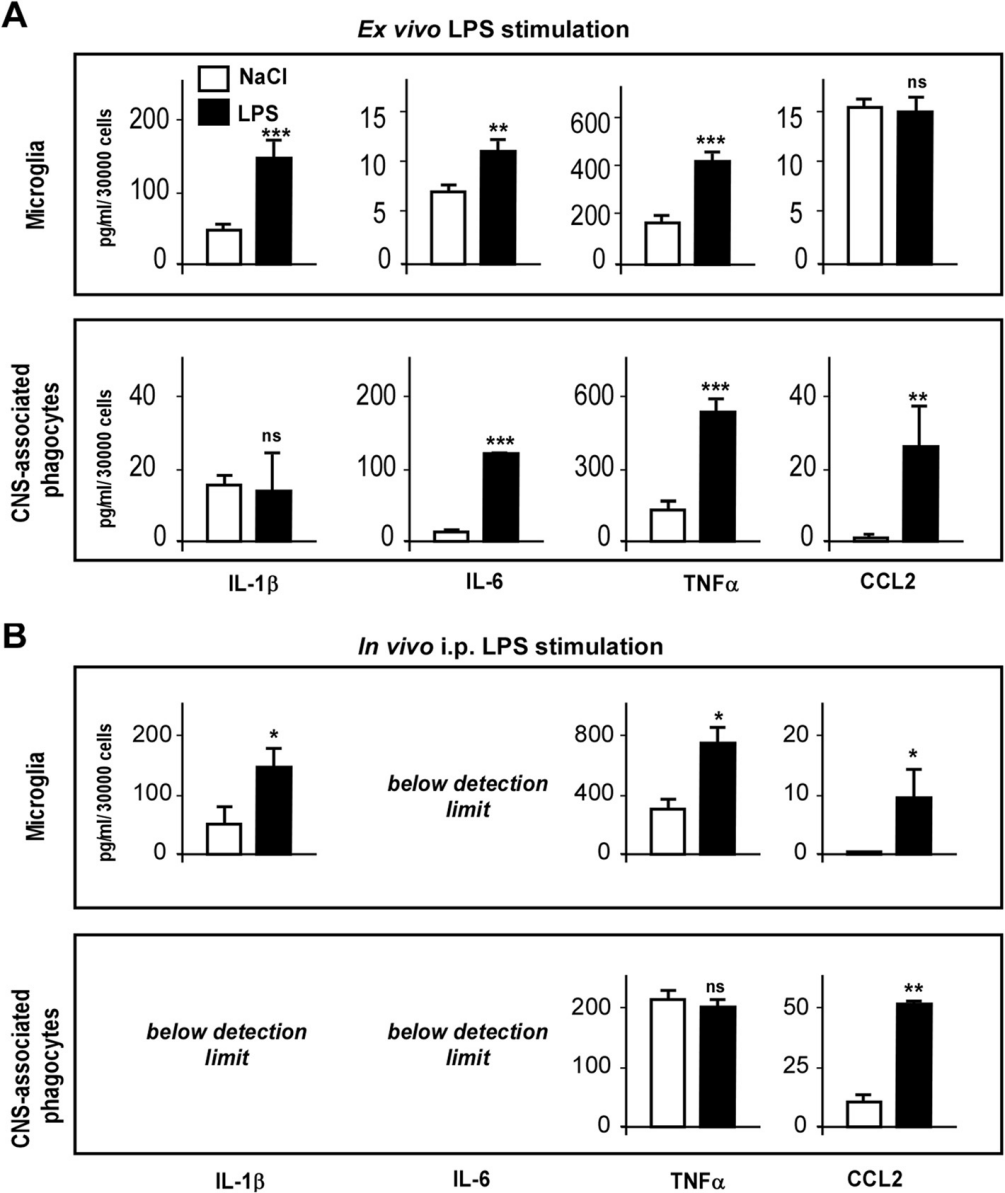
**Lipopolysaccharide promotes M1 pro-inflammatory cytokines and chemokine production by microglia and central nervous system-associated phagocytes sorted from mouse brain.** Microglia and central nervous system (CNS)-associated phagocyte enriched cell populations were sorted according to CD11b and CD45 expression patterns, resuspended and incubated in culture medium for an additional 24 hours. Pro-inflammatory cytokines IL-1β, IL-6, TNFα and CCL2 chemokine were quantified by cytometric bead array. **(A) ***Ex vivo* stimulation with 0.5 μg/ml lipopolysaccharide (LPS) (black bars) compared to saline control (white bars). **(B) ***In vivo* stimulation by LPS injections in mice as described in Methods (black bars) compared to saline injections (white bars). Bars represent the mean ± SEM. *P < 0.05, **P < 0.01, ***P < 0.005 versus saline control; n = 3-8. i.p., intraperitoneal.

To delve deeper into the understanding of LPS direct effects on microglia and CNS-associated phagocyte activation, we identified these two populations in control mouse brain cell suspension according to CD11b and CD45 marker expression levels as previously shown. We separately sorted them from untreated mouse brains and stimulated them *ex vivo* with LPS for 24 hours. CBA analysis of secretion media revealed that LPS induced dramatic increases in IL-6 and TNFα release by both microglia and CNS-associated phagocyte populations (Figure 4A). Interestingly, LPS did not elicit the same effects on IL-1β and CCL2 production in these two cell populations. Indeed, whereas *ex vivo* LPS treatment induced a strong increase of IL-1β production by sorted microglia, it did not alter IL-1β release by CNS-associated phagocytes (Figure 4A). On the contrary, LPS treatment promoted CCL2 production by sorted phagocytes but not by microglia (Figure 4A). These experiments allowed us to emphasize distinct sensitivity and responsiveness of microglia and CNS-associated phagocytes to direct *ex vivo* stimulation by LPS.

It is clear that peripheral administration of LPS promotes a neuroinflammatory response; however, how peripheral LPS induces its effects on the brain is not exactly known. In order to compare direct effects of LPS on microglia/phagocytes with indirect effects of peripherally administered LPS, we sorted microglia and CNS-associated phagocytes from mouse brains 24 hours after saline or LPS i.p. injection, cultured them for 24 additional hours without *ex vivo* restimulation, and analyzed secretion of cytokines and chemokines. Microglia sorted from the brain of LPS-treated mice retained their inflammatory characteristics after sorting as they secreted significantly more IL-1β, TNFα and CCL2 than control microglia from the brain of saline-treated mice (Figure 4B). IL-6 release by sorted microglia was not significantly affected by peripheral LPS treatment and stayed below the detection range in both conditions (Figure 4B). Analysis of secretion media from sorted CNS-associated phagocytes revealed that LPS only affected CCL2 release by those cells, while other cytokine secretions remained unchanged or below detection range (Figure 4B). These results show that LPS administered peripherally does not produce the same pro-inflammatory effects on microglia and brain phagocytes as when it is administered directly in culture. These data suggest the existence of probable intermediates between LPS and microglia/phagocytes in the brain and highlight the predominant role of the CCL2 chemokine in neuroinflammation induced by peripheral LPS challenge. This hypothesis is further reinforced by results showing that intracerebroventricular injection of LPS caused a massive and selective increase of IL-6 and CCL2 levels by sorted microglia and CNS-associated phagocytes, respectively, similarly to direct *ex vivo* stimulation but differently from *in vivo* stimulation (data not shown).

### CCL2 hyperpolarized serotonergic dorsal and median raphe neurons in mouse midbrain slices

Although a dysfunction of the serotonergic system alone cannot explain the full pathophysiology of depression, it is considered one of the key factors in this disease. Here we examined whether pro-inflammatory chemokine CCL2 could alter serotonergic neuronal activity in dorsal and median raphe nucleus, a major source of serotonergic input to the forebrain.

**Figure 5:**
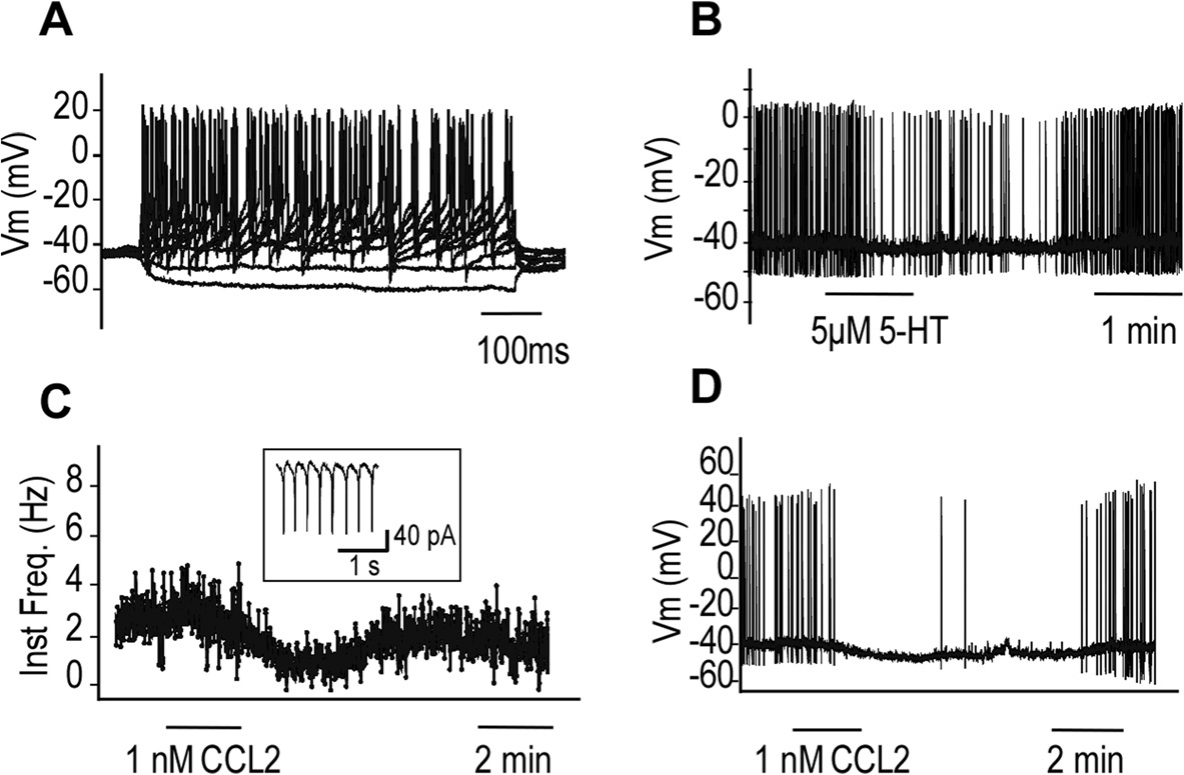
**CCL2 hyperpolarizes serotonergic dorsal and median raphe neurons in mouse midbrain slices. (A)** Example of recordings of the membrane potential of a presumed serotonergic neuron from the raphe nucleus recorded in current-clamp in response to increasing current pulses from −20 pA to 70 pA with an increment of 10 pA. **(B)** Spontaneous action potential discharge of a presumed sertonergic neuron recorded in current clamp and its response to the application of serotonin in the bath (5-HT; as indicated by the bar). **(C)** Graph presenting the instantaneous frequency of action potential discharge of a presumed serotonergic neuron recorded extracellularly in loose patch. The inset presents an example of recording obtained. **(D)** Spontaneous action potential discharge of a presumed serotonergic neuron recorded in current clamp and its response to the application of 1 nM CCL2 in the bath (as indicated by the bar).

In transverse acute brain slices of mesencephalon, we recorded neurons using the patch-clamp technique at the level of the dorsal and median raphe nucleus. Neurons were first recorded in cell-attached mode, and the firing frequency was determined when the neurons where spontaneously active. The firing frequency ranged from 0.2 to 4 Hz with a mean of 1.22 ± 0.35 Hz (n = 7). When possible, we switched to whole cell in the current-clamp mode and determined the membrane potential of the neurons. The membrane potential of the presumed serotonergic neurons ranged from −60 to −40 mV with an average of −55.00 ± 3.27 mV. Neurons were then recorded in the current-clamp mode. They were first characterized by their responses to current steps of increasing amplitude (Figure 5A). Serotonergic neurons express 5HT-1a receptors which, upon activation by serotonin, induce the activation of a G protein-coupled inwardly-rectifying potassium channel and subsequent hyperpolarization [56]. The recorded neurons were further identified as serotonergic when they were hyperpolarized and/or when their firing discharge was decreased by 5-HT (5 μM), applied at the end of the recording (Figure 5B).In presumed serotonergic neurons, we observed that CCL2 (1 nM) induced a decrease in the instantaneous firing frequency of neurons recorded in extracellular mode (Figure 5C) as well as current-clamp mode (Figure 5D) in 6 out of 7 neurons. This decrease in firing frequency was accompanied by a hyperpolarization ranging from −2 to −30 mV (with a mean of −10.50 ± 4.06 mV, n = 6); that is of a similar magnitude to that obtained following the application of 5 μM serotonin (compare Figure 5B and D). The application of CCL2 induced a decrease in the membrane resistance of the neurons (n = 5, data not shown), suggesting that CCL2 induced the opening of G-protein activated potassium channels in the membrane. Overall, CCL2 decreased the firing of 5-HT neurons by hyperpolarizing them and by decreasing their membrane resistance.

## Discussion

Previous studies indicate that peripheral immune challenge induces depressive-like behavior in rodents and humans (for review, see [57]). This corresponds to the induction of neuroinflammation, characterized by overproduction of pro-inflammatory cytokines, mainly IL-1β and TNFα, within the brain, resulting in HPA axis perturbations, microglia activation, peripheral immune cell infiltration, hippocampal neurogenesis inhibition and serotonergic transmission alteration (for review, see [58]), but the relative contribution of cytokines/chemokines and brain-resident or brain-infiltrating immune cells remained unclear.

In this study, we demonstrate that acute systemic LPS challenge elicits a pro-inflammatory signature in different brain areas involved in depressive processes (such as the hypothalamus and hippocampus). Although LPS is known to induce the synthesis and release of many pro-inflammatory mediators, which cytokines are involved in the LPS-induced brain mediated responses needed to be clarified. We show that the profile of cytokines and chemokines produced within the brain after LPS injection corresponds to a M1 classical type of activation of microglia/macrophages. Interestingly, we also found a highly significant upregulation of IL-4Rα expression, a specific marker of the M2c “classical deactivation” state, on the surface of both microglia and CNS-associated monocytes. This is in agreement with publications showing that the simultaneous increase of markers of both classical activation and classical deactivation triggered by LPS is consistent with the proposed M2b monocyte phenotype [53,59,60]. However, unlike the results of Fenn and colleagues [53], we did not detect any increase in the LPS-treated brain of the anti-inflammatory cytokine IL-10 (data not shown). In conclusion, we can speculate that IL-4Rα may be upregulated on microglia and CNS-associated monocytes in order to promote a microglial phenotype permissive to tissue repair and the resolution of inflammation in response to increased levels of IL-4 [59,60].

The role of microglia in neuroinflammatory mechanisms and neurodegenerative diseases often shows gaps or inaccuracies regarding the discrimination between microglial cells, resident macrophages of the brain, and monocytes infiltrating the CNS in pathological conditions. In this study, we performed detailed flow cytometry analysis to distinguish these cell populations and decipher their respective sensitivity and responsiveness to a peripheral LPS challenge. With this technique, we show that systemic LPS promotes an important increase in inflammatory monocytes within the brain. Our results do not exclude the possibility of a proliferation of microglia and/or CNS-resident macrophages; however, this might be a minor event as compared to the massive migration of inflammatory monocytes from the periphery, at least 24 hours post-infection. Upregulation of the CD45 marker on the surface of microglia has been described. This could lead to the misidentification of microglia and CNS-associated monocyte populations in mice treated with LPS. However, it is unlikely that microglia were confused with inflammatory monocytes or neutrophils because microglia express neither Ly6G nor Ly6C (data not show). One can then imagine that some of the activated microglia overexpressing CD45 could be confused with CNS-associated monocytes. However, flow cytometry analysis revealed that microglia had a smaller size compared with monocytes and, although they displayed elevated CD45 levels and increased size and granularity in inflammation conditions, they could still be distinguishable from monocytes (data not shown and [61]).

The choroid plexus epithelium constitutes the structural basis of the blood-cerebrospinal fluid barrier. Interestingly, the selective increase of TNFα mRNA that we observe in this structure (see Table 1) may indicate alterations in the choroid plexus epithelial cell barrier [62] that could explain the infiltration of peripheral immune cells upon LPS treatment. It is noteworthy that this modification of choroid plexus permeability did not result in the migration of non-specific immune cells within the brain. Indeed, no neutrophils, T or B lymphocyte entry was observed 24 hours after LPS injection, in spite of the increase in IL-1β that we observed, which was previously described to induce the infiltration of leukocytes and neutrophils within the brain, although in a very different type of experimental conditions [63].

Direct LPS action on choroid plexus or BBB permeability is one of the possible mechanisms that have been proposed to explain LPS effects on the CNS. Alternatively, LPS could act outside the BBB by stimulating afferent nerves, acting at circumventricular organs, and indirectly altering BBB permeability and functions. LPS could also promote a general inflammatory response in the periphery, leading to modified immune cell and cytokine/chemokine profiles that could have an impact on the brain inflammatory status through microglia and CNS-associated monocytes and immune cell infiltration. Recent data from Banks and Erickson showed that brain uptake of circulating LPS was negligible and that most effects of peripherally administered LPS were likely mediated through LPS receptors located outside the BBB [64]. However, this conclusion is difficult to reconcile with our results showing a marked increase in the expression of TLR4 on microglia cells after systemic injection of LPS.

The chemokine CCL2 is known to facilitate recruitment of Ly6C^high+^ inflammatory monocytes, expressing high levels of CCR2, to lesion sites outside the CNS [65]. Here we show that the increase of CCL2 mRNA and protein amounts in the brain after systemic LPS is associated with an upregulation of CCR2^+^ expressing microglia and inflammatory monocytes, suggesting that cerebral CCL2 could also be responsible for peripheral immune cell migration into the brain in neuroinflammatory conditions. Although we did not demonstrate that these inflammatory monocytes do infiltrate brain parenchyma, it would be interesting to investigate whether such migration and/or infiltration would have beneficial or deleterious effects in the CNS [66]. The results obtained in this study answer, at least partially, this question. Indeed, the precise identification by flow cytometry of microglia and CNS-associated phagocytes allowed us to develop an efficient technique of selective cell sorting of these cell populations that we maintained in culture for 24 hours in order to analyze the cytokines and chemokines that they release, which are likely to reflect what they secrete *in situ*. One cannot exclude the possibility that the brain cell suspension protocol, cell sorting procedure and culture conditions might have modified microglia and monocyte features; however, this is very unlikely as we demonstrate that microglia sorted from control mice still have the ability to activate under the influence of *ex vivo* stimulation by LPS and that significant and coherent differences between cells sorted from brains of saline- and LPS-treated mice still are detectable. Moreover, it should be noted that such techniques are routinely used by immunologists to study inflammatory features of cytokines and chemokines secreting immune cells. Interestingly, our results highlight that microglia and CNS-associated phagocytes should absolutely be distinguished when studying neuroinflammatory processes as these two cell populations do not have the same sensitivity and responsiveness characteristics towards *in vivo* or *ex vivo* stimulation with LPS. Indeed, our study highlights the fact that if an *in vivo* treatment with LPS elicits IL-1β, TNFα and CCL2 release by microglia, it might only trigger CCL2 release by CNS-associated phagocytes. This indicates that CCL2 released in the CNS by microglia would account for the chemoattraction of circulating CCR2^+^ inflammatory monocytes. These cells also secrete CCL2, thus elevating even more the cerebral CCL2 level, possibly creating an amplification loop that could explain the magnitude of the infiltration of these cells into the brain. In brain immunity, blood monocyte-derived macrophages may have similar functions in brain repair to the ones they have in peripheral injuries such as skin wounds and myocardial infarcts [67]. Indeed, monocytes and their tissue-related macrophages are key regulators in tissue healing processes, which is a phenomenon that is schematically biphasic. In infarct, for example, Ly6C^high^ pro-inflammatory CCR2^+^ monocytes attracted to the damaged heart by release of CCL2 dominate on days 1 to 4 (phase 1) and promote digestion of infarcted tissue and removal of necrotic debris, whereas reparative Ly6C^low^ CCR2^−^ monocytes dominate during the resolution of inflammation (phase 2) and propagate repair. Such mechanisms are thought to also occur in the case of brain damage. Microglia and astrocytes produce CCL2 upon pro-inflammatory stimulus (systemic LPS in the present study), responsible for recruitment of blood Ly6C^high^ pro-inflammatory CCR2^+^ monocytes within 24 hours (as shown by our results). We did not investigate brain-associated monocyte phenotype later on after LPS injection; however, it is likely that after this early pro-inflammatory phase 1, a following tissue repair phase 2 of active resolution of inflammation would occur.

Our results points to CCL2/CCR2 signaling as a crucial element in the development of neuroinflammation in the first days following a peripheral infection. This is supported by a previous study by Thompson and colleagues showing that CCL2^−/−^ mice show a downregulation of brain pro-inflammatory cytokine production and decreased activation of brain immune cells after systemic LPS treatment compared to wild-type CCL2^+/+^ mice, despite a higher level of pro-inflammatory cytokines in the serum [68]. Additionally, Wohleb and colleagues showed that anxiety-like behavior associated with repeated social defeat was linked to CCR2-mediated recruitment of peripheral monocytes into the brain [69,70].

Although complex emotional states cannot be reduced to the imbalance of a single neurotransmitter, a prominent participation of 5-HT in depression and anxiety is generally acknowledged [71]. From our results on the hippocampus and hypothalamus and the results from the literature, it is likely that the increased CCL2 level in response to systemic LPS injection is widespread in all areas of the brain parenchyma, including the median and dorsal raphe nucleus. We thus examined whether CCL2 could directly regulate 5-HT release by serotonergic neurons of the median and dorsal raphe nucleus. Our results show that CCL2 induced a hyperpolarization and a decrease in firing of serotonergic neurons of the median and dorsal raphe nucleus. These results are in agreement with the effects of other chemokines on serotonergic neurons [72,73] and extend to 5-HT neurons the neuromodulatory effects of CCL2 already demonstrated in other neuronal populations such as neurons of the dorsal root ganglion [74], dopaminergic neurons of the substantia nigra [75] or melanin concentrating hormone neurons (unpublished data of Alice Guyon). These effects of CCL2 on 5-HT neurons should lead to a reduction of 5-HT release in projection areas such as the amygdala and hippocampus, two regions implicated in the regulation of emotional expression and memory processes affected in major depression. However, one can hypothesize that in our experiments CCL2 may act on neurons through CCR2-independent pathways. This hypothesis cannot be totally ruled out inasmuch it has been recently shown that CCL2, acting on benzodiazepine sites, can modulate the GABA-evoked currents, depending on the subunit composition of GABA(A) receptor [76].

## Conclusions

In conclusion, our data suggest that during endotoxemia caused by acute systemic LPS challenge, CCL2 is a crucial element for full activation of the brain resident immune cells, production of endogenous brain inflammatory mediators, inflammatory immune cell infiltration and reduction of 5-HT release by serotonergic neurons; all of these elements being linked to the establishment of depressive behaviors.

More generally, this study brings new elements emphasizing the importance of neuroimmunopsychopharmacology aimed at studying the functional interactions between living conditions, the central and peripheral nervous system, the immune system and the consequences of this dialogue on physical and mental health.

## Abbreviations

5-HT: serotonin; BBB: blood–brain barrier; CBA: Cytometric Bead Array; CCL2: chemokine (C-C motif) ligand 2; CCR2: C-C chemokine receptor type 2; CNS: central nervous system; DA: dopamine; FSC: Forward Scatter; HPA: hypothalamus-pituitary-adrenal; Iba-1: ionized calcium binding adaptor molecule 1; Ig: immunoglobulin; IL: interleukin; i.p.: intraperitoneal; LPS: lipopolysaccharide; NE: norephinephrine; PBBS: phosphate/bicarbonate buffered solution; PBS: phosphate-buffered saline; PCR: polymerase chain reaction; SSC: side scatter; TLR4: Toll like receptor 4; TNF: tumor necrosis factor.

## Competing interests

The authors declare that they have no competing interests.

## Authors’ contributions

JCh and APP conceived the study. JCa, JCh and APP developed the brain immune cell preparation, characterization and cell sorting techniques. JCa carried out the flow cytometry, cell sorting, cytometric bead array experiments and analysis. AG performed electrophysiological experiments. APP performed quantitative PCR analysis, coordinated the study, analyzed the data and drafted the manuscript with the assistance of the other authors. CH provided helpful advice and fruitful discussion. All authors have read and approved the final version of the manuscript.
